# Possible connection between intestinal tuft cells, ILC2s and obesity

**DOI:** 10.3389/fimmu.2023.1266667

**Published:** 2024-01-12

**Authors:** Hong Yang, Yu-Xing Huang, Pei-Yu Xiong, Jin-Qian Li, Ji-Lan Chen, Xia Liu, Yan-Ju Gong, Wei-Jun Ding

**Affiliations:** ^1^ Chengdu University of Traditional Chinese Medicine, Chengdu, China; ^2^ Hospital of Chengdu University of Traditional Chinese Medicine, Chengdu, China

**Keywords:** obesity, tuft cell (TC), group 2 innate lymphoid cell (ILC2), TC-ILC2 circuit, intestinal remodeling, immune homeostasis

## Abstract

Intestinal tuft cells (TCs) are defined as chemosensory cells that can “taste” danger and induce immune responses. They play a critical role in gastrointestinal parasite invasion, inflammatory bowel diseases and high-fat diet-induced obesity. Intestinal IL-25, the unique product of TCs, is a key activator of type 2 immunity, especially to promote group 2 innate lymphoid cells (ILC2s) to secret IL-13. Then the IL-13 mainly promotes intestinal stem cell (ISCs) proliferation into TCs and goblet cells. This pathway formulates the circuit in the intestine. This paper focuses on the potential role of the intestinal TC, ILC2 and their circuit in obesity-induced intestinal damage, and discussion on further study and the potential therapeutic target in obesity.

## 1 Introduction

Obesity is a worldwide pandemic and a major risk for chronic diseases, including diabetes and cardiovascular disease. The prevalence of obesity has been increasing rapidly worldwide due to changes in lifestyle and diet. Obesity is considered as a typical metabolic syndrome characterized by systemic low-grade chronic inflammation (1). The close interactions between obesity and the gut microbiota and immunity (2) have recently been revealed, but the underlying mechanism has not yet been fully elucidated.

TCs line the epithelial mucosa, such as the intestine, airway and taste bud (3, 4). Although they were discovered more than six decades ago, their exact roles in health remain mysterious. Under homeostatic conditions, a small number of TCs serves as the monitor in the intestines, detecting and responding to various stimuli through the use of succinate (5), sweet and bitter taste receptors. While investigating ulcerative colitis (UC), the researchers noticed a reduction in TCs along with inflammation and tissue damage. However, when they treated UC mice with succinate, they observed an improvement of chronic intestinal inflammation, accompanied by an increase in the number of intestinal TCs (6). TCs are characterized by their apical microvilli and orchestrate the mucosal immune response (7), such as parasite immunity (8–10). Notably, intestinal TCs are unique cells that secrete IL-25 (10), the key activator of ILC2s. In response to IL-25, enteric ILC2s produce type 2 cytokines, including IL-4, IL-5, IL-9 and IL-13, which promote the ISCs differentiation into TCs, thus formulating the circuit (10). The TC-ILC2 circuit is regulated by feed-forward signaling including IL-25, IL-4 and IL-13 (11, 12), which has been shown to regulate intestinal remodeling and homeostasis in response to diet (12). Arora (13) indicated that TCs may be involved in modulating whole-body energy metabolism. ILC2s and their cytokines in the small intestine can also contribute to inflammation and insulin resistance (14). Further understanding of the potential connection between intestinal TC, ILC2 and obesity needs to be conducted. Herein, we review the connection mechanism of the intestinal TC, ILC2 and their circuit in obesity-associated inflammation through intestinal metabolic homeostasis.

## 2 Intestinal TCs in obesity process

### 2.1 Distribution, morphology, heterogeneity and origin of intestinal TCs

**Figure 1:**
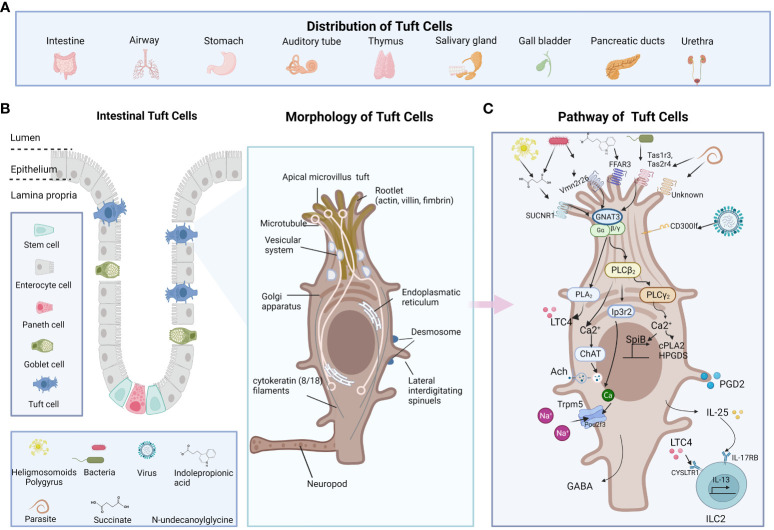
The distribution, morphology, and functions of TCs. **(A)** TCs present in the columnar epithelium of hollow endoderm-derived organs including trachea, thymus, stomach, small intestine, and urethra in mammals **(B)** The location and morphology of the TCs are shown in the middle. TC is characterized by its long and compact microvilli, which are connected to a broad network of rootlets that consists of actin, villus, filaments, and microtubules that hold the direction from apical to basal. **(C)** The receptor of TCs including Tas1Rs/Tas2Rs, SUCNR1, FFAR3, Vmn2r26, CD300lf, and some unknown. The pathway of TCs including GNAT3-Plcβ2-Ip3r2-TRPM5, GPCR-PLCγ2)-Ca(2+) signaling axis and et al. The figure was created by Biorender.

TCs are mostly present in the columnar epithelium of hollow endoderm-derived organs such as the trachea, thymus, stomach, small intestine, and urethra in mammals (15). Both the small and large intestines contain TCs, with the highest number at the proximal small intestine (16). Cholecystokinin, peptide YY- and glucagon-like peptide-1 (GLP-1) positive endocrine cells are found close to TCs (16). More than 60% of TCs in the small intestine are found in contact with nerve fibers, indicating that TCs are modulators of intestine movements (16) (**Figure 1A**).

The structure of TCs was first described in 1956 by Rhodin and Dalhamn (17). The “tuft” or “brush” comes from its unique type of brush border, and the apical microvilli of TCs extend into the gut lumen (18). The TC is characterized by its long and compact microvilli, which are connected to a broad network of rootlets that consists of actin, villus, filaments, and microtubules that hold the direction from apical to basal relative to neighboring cells (19–22). The cytoskeleton is largely composed of intermediate actin filaments (18, 23). Cytoplasmic spinules are a special part of TCs that extend from one lateral of the TC border into the adjacent cells, reaching their nuclei, which could be the route for the transport of molecular cargo wrapped with IL-25 or acetylcholine to the adjacent cells (18, 23, 24). These lateral interdigitating spinules appear on TCs ranging from just below the apical tight and adherent junctional complex to the nuclear level. With no secretory vesicles extending down the basal lamina, the basal area of TCs develops protrusions that resemble neuropods, indicating paracrine communication via neuropods (11) (**Figure 1B**).

By utilizing the single-cell sequence approach, Haber and his colleagues defined two subtypes of TCs, tuft-1 and tuft-2, in the intestine (25). Montoro also found the two subtypes in the mouse tracheal epithelium (26). Tuft-1 signature genes are correlated with neuronal development, whereas Tuft-2 cells are enriched for immune-related genes. SH2 Domain Containing 6 (*Sh2d6*) and G protein-coupled receptor (GPCR) vomeronasal type-2 receptor 26 (*Vmn2r26*) have been proposed as exclusive markers for tuft-2 cells (27), while tubulin polymerization-promoting protein family member 3 (*Tppp3*) is primarily expressed in tuft-1 cells (27). Besides, Tuft-2 cells express significantly higher levels of the Th2-promoting cytokine, thymic stromal lymphopoietin (TSLP) (25, 28). Markers including Arachidonate 5-lipoxygenase (*Alox5*), Cytochrome c oxidase 1 (*Cox1*), Cytochrome c oxidase 2 (*Cox2*), Hematopoietic Prostaglandin D Synthase (*Hpgds*), HOP Homeobox (*Hopx*), and phosphoserine epidermal growth factor receptor (*p-Egfr*) (29–31) exist in both subtypes. POU class 2 homeobox 3 (*Pou2f3*) is a crucial transcription factor for TC development, showing a shared ancestry with TCs in the gastrointestinal tract and lingual taste buds (9). In addition, growth factor-independent 1B (*Gfi1b*) is identified as TC-specific master regulators (32, 33). Both markers are expressed constitutively in all TCs (7). However, tuft-1 and tuft-2 cells both express IL-25 (25). **Table 1**.

**Table 1:** The biomarkers of intestinal tuft cells.

Marker	Name	Description of Proposed Function	Reference
Chemosensory			
*Trpm5*	Transient receptor potential cation channel subfamily M member 5	The chemosensory functional cation channel induces a type 2 response	(34, 35)
*Gnat3*	Guanine Nucleotide-binding protein G(t) subunit α3	Activation of calcium efflux which triggers the release of signaling molecules such as IL-25	(36)
*Chat*	choline acetyltransferase	Chat catalyzes the transformation of acetyl-CoA from mitochondria into ACh in the cytoplasm	(37)
*Sh2d6*	SH2 Domain Containing 6	A signature marker for CD45^+^ Tuft-2 cells related to immune response	(10, 27)
*Vmn2r26*	vomeronasal type-2 receptor 26	High expressed in Tuft-2 cells, which is responsible for detecting the bacterial metabolite.	(27)
*Tppp3*	Tubulin polymerization-promoting protein family member 3	*Tppp3* is a unique marker gene for Tuft-1 cells	(27)
Morphology			
*Dclk1*	Doublecortin-like kinase 1	Regulate tissue regenerative responses and enhance epithelial repair responses.	(38)
*CK18*	Cytokeratin 18	Contribute to the cytoskeletal make-up	(39)
*α-tubulin*	Acetylated α-tubulin	Part of microtubule bundles	(7)
*Hopx*	HOP Homeobox	Predicted to enable DNA binding activity. Located in nucleus. Is expressed in several structures	(34)
*Siglec-F*	Sialic acid-binding Ig-like lectin F	A cell surface lectin whose expression is evident in intestinal tuft cells	(40)
Metabolic activity			
*Alox5*	Arachidonate 5-Lipoxygenase	Enables arachidonate 5-lipoxygenase activity	(29–31)
*Cox1*	Cytochrome c oxidase 1	Prostaglandin synthesis and damage recovery	(29–31)
*Cox2*	Cytochrome c oxidase 2	Prostaglandin synthesis and damage recovery	(29–31)
*Hpgds*	Hematopoietic Prostaglandin D Synthase	Prostaglandin synthesis and damage recovery	(41)
*p-Egfr*	Phospho-Epidermal growth factor receptor	The enzyme which regulates the development and differentiation	(29, 42)
Transcription factor			
*Sox9*	SRY-Box Transcription Factor 9	Regulate the development, cell-fate determination, and differentiation	(41)
*Pou2f3*	POU Class 2 Homeobox 3	*Pou2f3* is an essential transcriptional factor for TCs development	(7, 32)
*Gfi1b*	Growth Factor Independent 1B Transcriptional Repressor	TCs specific master regulators for differentiation	(43)
*Atoh1*	Atonal bHLH Transcription Factor 1	TCs specific master regulators for differentiation	(30)
Cytokine			
IL-25	Interleukin-25	Solely produced by tuft cells in the intestine	(12)

Since the intestinal epithelium has rapid turnover and vigorous proliferation, and ISCs renew every 4-7 days, it is essential canonical Wnt signals combined with TGFβ/BMP pathways proceed unperturbed to maintain the integrity of the intestinal epithelial barrier (44). ISCs identified as leucine rich repeat-containing G protein coupled receptor-5 (Lgr5) positive cells, which are responsible for both renewal and regeneration processes and are located at the base of crypts (45). TCs are relatively rare in the intestine and account for 0.4% to 2.3% of the total epithelial cells in the murine intestinal epithelium (25). Unlike other epithelial cells, differentiated intestinal TCs still express Lgr5 (29).

The origin of intestinal TCs has been a controversial topic for years. Gerbe (30) first demonstrated that mature TCs are derived from Lgr5^+^ epithelial stem cells and require atonal bHLH transcription factor 1 (*Atoh1*) to differentiate, identifying them as the secretory lineage. However, some researchers hold a different opinion that doublecortin-like kinase 1 (DCLK1) positive TCs are derived from Lgr5^+^ stem cells and require neural input for survival in the nonsecretory lineage rather than the columnar lineage (43, 46). Herring (47) used p-Creode analysis of single cells in the intestine with computational and experimental methods to indicate that TCs were possibly *Atoh1*-independent secretory lineage in the small intestine. while in the colon, TCs arise from an *Atoh1*-driven alternative developmental program.

### 2.2 Functional pathways of intestinal TCs

Although TCs were discovered as a unique cell type more than sixty years ago, their function was not fully explored. Sensory and secretory functions were the known main functions of TCs, moreover, they are usually correlated.

TCs exhibit significant similarities with sensory cells. They possess characteristic apical microvilli that are positioned toward the luminal interface, like the tongue’s taste buds. This positioning facilitates access to chemosensory nerve endings. A variety of apical and basolateral receptors allow TCs to detect different parasites, bacteria, viruses or their metabolites (8, 48, 49). TCs taste signal receptors, including Type 1 transduces signals (Tas1Rs) for sweet/umami substances and type 2 for bitter substances (Tas2Rs) (50, 51), succinate receptor 1 (SUCNR1) (5, 12), and free fatty acid receptor 3 (FFAR3) (52) are heterotrimeric guanine nucleotide-binding protein G-coupled receptors (GPCRs). The activation of these distinct receptors triggers a shared signal transduction pathway that involves *Gnat3*, phospholipase Cβ2 (*Plcβ2*), inositol triphosphate receptor type 2 (*Ip3r2*), and *Trpm5* a monovalent specific, nonselective cation channel for Na^+^, K^+^, and Cs^+^ ions instead of Ca2^+^ ions (53). *Pou2f3* is the master regulator for the generation of *Trpm5*-expressing chemosensory TCs (54). These *Trpm5*-expressing TCs are responsible for helminth infection in the gut (8). However, TCs make use of *Ip3r2* to regulate cytosolic calcium and Trpm5 activity in response to Trichinella spiralis infection in a small intestinal organoid, while Trpm5 enhanced the release and hyperplasia of IL-25 (51). The loss of activating transcription factor 5 (*Atf5*) enhanced the TC-ILC2 circuit by promoting TC differentiation in response to parasitic infections (55). While it is controversial, *Heligmosomoides polygyrus* seems to block the effects of IL-4 and IL-13 and inhibit the gene expression of TCs both *in vitro* and *in vivo (* 56). As for the intestinal antimicrobial immunity, tuft-2 senses bacterial metabolite N-undecanoylglycine, then *Vmn2r26* engages activated G-protein-coupled receptor-phospholipase C gamma2 (GPCR-PLCγ2)-Ca^(2+)^ signaling axis, which produces prostaglandin D2 (PGD2). PGD2 further enhances antibacterial immunity (27). Moreover, *Vmn2r26* signaling also promotes the expression of Spi-B transcription factor, which promotes the expansion of Tuft 2 cells (27). Expressed on the intestinal TCs, CD300lf is identified as a murine norovirus (MNV) receptor, promoting MNV infection *in vivo* (57). Denatonium, as a bitter substance, is reported to increase intracellular calcium levels in colonic TCs (58). SUCNR1 is a typical receptor that reacts to succinate secreted by symbiotics and helminths (5). FFAR3, another TC receptor, is reported to be sensing indolepropionic acid (IPA), restoring IL-25 function, preventing gut leakage and inhibiting systemic inflammation (52). Interestingly, most reports indicate that multiple taste-associated GPCRs initiate intestinal immunity in TCs (**Figure 1C**).

TCs are important intestinal secretory cells. The majority of intestinal TCs generate a variety of paracrine and endocrine cytokines, including IL-25 (8), ACh (41), eicosanoids, TSLP and β-endorphins (25). TCs release IL-25 upon helminth infection and activate ILC2s to secrete IL-13, while IL-13 promotes the ISCs differentiation into TCs and goblet cells (10). The IL-25-dependent TC circuit requires macrophage migration inhibitory factor (*Mif*) (59). TCs express choline acetyltransferase for acetylcholine (ACh) synthesis, leading to the activation of *Trpm5* during helminth infection (60). TCs also rapidly synthesize leukotrienes and other eicosanoid derivatives to stimulate competent cells (51, 61). Moreover, TCs are responsible for opioid (62), prostanoid and β-endorphin production (30), which are associated with analgesic effects as well as basic intestinal physiology including intestinal motility, secretion and nutrition absorption (**Figure 1C**).

### 2.3 Intestinal TCs in obesity

The intestines play a crucial role in the absorption of nutrients and the elimination of waste materials (63). Excess fat uptake in a Westernized diet is associated with an increased incidence of inflammatory bowel disease, obesity and food allergies (64). High-fat diet (HFD) impairs the intestinal immune system, making it hypersensitive to epithelial damage (65). Besides, it induces the imbalance in the gut microenvironment and active epithelial mucosa cells, including TCs (66).

It is controversial that TCs number varies when the mice are fed with HFD. Widmayer reported that 3 weeks of HFD supply increased the gastral surface mucous cells, mucosal mast cells, and TCs (67), and the expansion TCs demonstrated active leukotriene (LT) secretion. Gastric TCs can quickly sense saturated long-chain fatty acids (LCFAs) through the apical receptors GPR120 (67). Then, it is likely that upon detecting saturated LCFAs, TCs may trigger inflammatory reactions that produce cysteinyl (cys) LTs and activate surface mucous cells and mucosal mast cells, thus regulating the function and integrity of the intestinal mucosa (67). This process could potentially play a role in triggering mucosal inflammation. However, Arora reported that HFD-induced obese mice present an overall decrease of TCs in the small intestine due to the inhibition of *IL-25* and *TSLP* mRNA expression (48). Additionally, Chen (52) and Sun (68) independently observed a reduction in TC population in mice subjected to long-time HFD, which coincided with a decrease in tight junction protein levels. However, TCs were both restored to normal levels following a weight loss intervention. It suggested that restoring the number of TCs in individuals affected by a HFD could potentially promote the restoration of intestinal barrier integrity. Aliluev (69) used single-cell sequencing on small intestinal crypt cells in nutrient-induced obese mice and found a decrease both in TCs and tuft progenitors. Mice fasted for 48 hours presented elevated levels of TCs after refeeding (29). However, a short-term HFD diet for 1, 3, or 7 d decreased the amount of TCs (70). Interestingly, the number of TCs exhibits considerable variation when they are subjected to HFD. It is plausible that the duration of HFD exposure, such as the 3-week HFD regimen employed by Widmayer (67) or the 9-week HFD regimen employed by Arora (48), may contribute to this difference.

Li reported that the succinate-SUCNR1 axis attenuates HFD-induced metabolic disturbance via activating type 2 immune responses and repairing intestinal barrier dysfunction (71). In mice infected with MNV, researchers discovered that intestinal TCs were targeted by MNV to activate mucosal immunity. This activation included various components such as B cell subsets, macrophages, and T cells, which collectively protected against type 1 diabetes in the mice (72).

The listed references provide evidence indicating a connection between disorders in taste cells and metabolic diseases, particularly obesity. Nevertheless, additional research especially the knockout of TCs or the key pathway is needed to further explore the role of TCs in these conditions.

## 3 ILC2s altered immunity in obesity

### 3.1 Origin, distribution, and function of ILC2s

ILC2s were recently found to be tissue-resident cells that lack recombination activating gene (RAG)-dependent rearrangement of antigen receptors, despite their particular lymphoid morphology compared to that of T and B cells (73). ILC2s were initially found in early infection of helminths (74). They are defined by biomarkers, including CD127 (IL-7Rα), CD161 (Klrg1) and CD294 (CRTH2) (75) (**Table 2**), which originate from the common lymphoid progenitor (CLP) in the fetal liver or bone marrow and migrate to peripheral tissues to develop into tissue-resident lymphocytes to combat specific types of infections (88, 89). CLPs are induced by *Gata3*, *Id-2* (90, 91) and the transcription factors *Gfi1* and *Bcl11b* to differentiate into ILC2s (92). ILC2s constitutively express high levels of GATA3 and ROR-α (90, 91), which are required for functional maturation and maintenance. The upstream regulator *Bcl11b* maintains *Gfi1* expression in mature ILC2s (92). ILC2s show the immune function of type 2 immunity, which is stimulated by extracellular parasites, food, microbes and allergens (93–95). IL-25, IL-33, and TSLP activate ILC2s and produce type 2 cytokines IL-4, IL-5, IL-9, IL-13 and amphiregulin, which contribute to pathogen defense, metabolic homeostasis, and tissue remodeling (49, 96–100). ILC2s are divided into two types: natural ILC2s (nILC2s) and inflammatory ILC2s (iILC2s). nILC2s respond to IL-33 and produce IL-9 (93), while iILC2s respond to IL-25 (101) and highly express killer cell lectin-like receptor G1 (KLRG1) (101) and promote their production of IL-13 (102).

**Table 2:** The markers of intestinal ILC2s.

Marker	Name	Description	Reference
CD25 (IL-2Rα)	Interleukin-2 receptorα	Showing percentages of ILC2s	(76)
CD45	CD45	CD45^+^ Lin^−^ cells, showing percentages of ILC2s	(77)
CD90 (Thy1)	Thy-1 cell surface antigen	Marker of ILC2s	(78)
CD127 (IL-7Rα)	Interleukin-7 receptor α	Favors ILC2 differentiation	(79)
CD161 (Klrg1)	Killer cell lectin-like receptor G1	Marker of mature tissue ILC2s	(75, 80)
CD294 (CRTH2)	chemoattractant receptor-homologous 2	Support ILC2 accumulation and migration in tissue	(81)
CysLT1R	Cysteinyl leukotriene receptor 1	Promote ILC2 production of IL-4, IL-5, and IL-13	(82)
IL-17RB	Interleukin-17 receptor B	The receptor of IL-25 essential to induce an expansion of ILC2	(83)
Ly6a (Sca-1)	Stem cells antigen-1	Typical ILC2 surface markers	(84)
ICOS	Inducible T-cell costimulator	Regulates ILC2 homeostasis by promoting proliferation and accumulation	(85)
NMUR1	Neuromedin U receptor 1	Production of innate inflammatory and tissue repair cytokines	(86)
ST2	suppression of tumorigenicity 2	Promote type 2 immune responses	(87)

### 3.2 Intestinal ILC2s are involved in obesity

HFD induces systemic low-grade chronic inflammation in animal models and humans. ILC2s work as a bridge linking epithelial cells and the immune system to promote immunity and metabolic homeostasis (103). In adipose tissue, ILC2s induce the type 2 cytokines IL-5 or IL-13 and increase eosinophils numbers, and M2 macrophages stimulate inflammation, which promotes immunity and metabolic homeostasis and curbs obesity (104, 105). In the intestine, Sasaki (14) used Il2rg^−/−^Rag2^−/−^ mice, which genetically lack all types of ILCs and are resistant to HFD-induced obesity, to reveal that intestinal ILC2s rather than WAT are more important in the development of obesity. The controversial results between the intestine and adipose tissue indicate that the immune function of ILC2s is likely dependent on the tissue/organ microenvironment. Administration of succinate in HFD-induced obese mice could enhance goblet cell production via activating type 2 immune responses in the small intestine (71). The expression of IL-4, IL-13, and IL-5 decreased in HFD-fed mice and was restored by berberine, indicating that ILC2s inhibit obesity-related intestinal inflammation (68). In obese human and mouse models, representative cytokines of ILC2s decreased and were rescued by IPA (52). In vitamin A deficient mice, dramatic expansion of IL-13 to produce ILC2 and resistance to nematode infection revealed that ILC2 might be the primary sensor of dietary stress, and innate type 2 immunity may represent a powerful adaptation of the immune system to promote host survival in the face of ongoing barrier challenges (89). The detailed effect of intestinal ILC2s on obesity still needs to be further investigated.

## 4 The TC-ILC2 circuit in obesity

### 4.1 Obesity intestinal microbial metabolites disorders potentially altered the circuit

Obesity and obesity-associated inflammation are closely correlated with dysbiosis in the intestinal microbiota (106). Short-chain fatty acids (SCFAs), including acetate, propionate and butyrate, are mostly produced by the gut microbiota (107). Mammalian cells produce succinate as an intermediate metabolite in the tricarboxylic acid cycle (TCA), yet microbes excrete succinate as a metabolic byproduct (108). Obese people have higher serum levels of succinate, which is associated with worse metabolic and specific intestinal microbiota (109). In a diet-induced obese model, succinate reduced body weight and promoted mitochondrial protein metabolism in brown adipose tissue (110). McKinley (29) noted that fasting and refeeding, as well as the introduction of microbiota to germ-free mice, caused dynamic changes in the quantity, composition and protein expression in TCs. Succinate is sufficient to promote the ISC to proliferate into TCs and goblet cells. However, at homeostasis, low levels of intestinal succinate cannot activate TCs (111). In response to the changes in TCs, intestinal microbial metabolites caused by HFD-induced obesity may affect the circuit, but further compelling evidence is required to support this claim.

### 4.2 The TC-ILC2 circuit potential function in obesity

**Figure 2:**
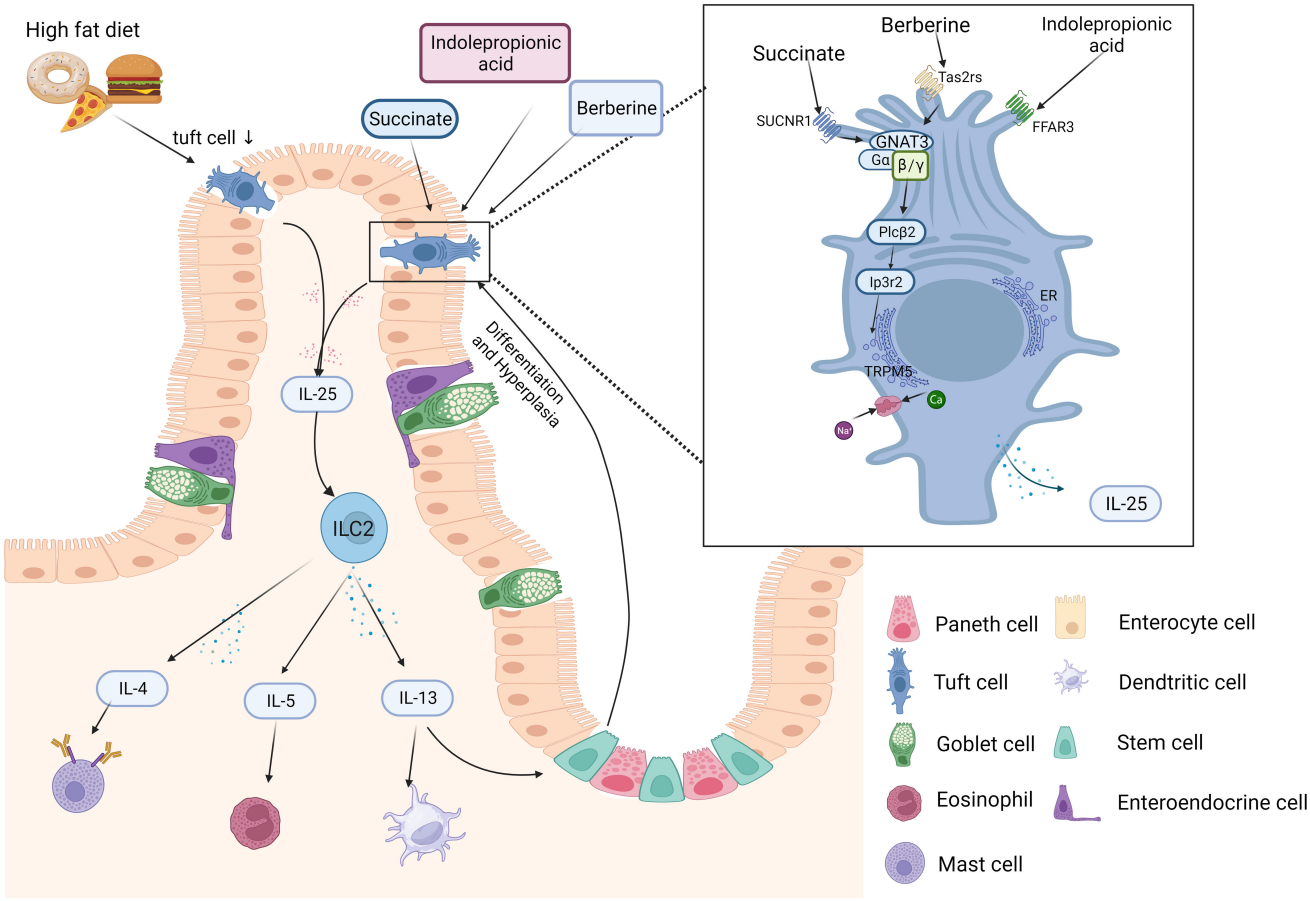
Schematic diagram of the intestinal TC-ILC2 circuit in obesity. Bacteria, metabolic byproducts (e.g., IPA and succinate), and helminths can be sensed by receptors on the surface of tuft cells (TCs) in the intestinal lumen. TC-derived IL-25 can induce Th2- and ILC2-secreted cytokines. Th2 cells mainly secrete IL-4, IL-5 and IL-13. ILC2s produce type 2 cytokines including IL-13 and IL-5. IL-13 can promote stem cell differentiation into TCs. IL-25 is the main molecule by which TCs “taste” metabolites (e.g., succinate) and activate signaling downstream pathways, including GNAT3 and TRPM5. The figure was created by Biorender.

The process of the circuit being affected can be explained as follows. The IL-25 receptor is the heterodimer of IL-17RB and IL-17RA. IL-25R is expressed in various respondent cells, including smooth muscle cells, basophils, eosinophils, and ILC2s throughout skin, brain, airway, and intestinal tissues (112–114). While TCs are the only cellular sources of IL-25 in the intestine (10), IL-25 induces ILC2s to produce type 2 immunity, which promotes the early immune response when infected (83). CysLTs and IL-25 collaborate to alert ILC2s to synthesize IL-4, IL-5 and IL-13 (61). IL-13 promotes Lgr5^+^ epithelial stem cells to differentiate into TCs or goblet cells, possibly by the Notch signaling pathway (115). In addition, IL-13 also promotes TC production and restores immune homeostasis and mucosal barriers (10). In response to dietary, the TC-ILC2 circuit regulates adaptive intestinal remodeling (12). However, this circuit is disrupted and downregulated *IL-25* and *TSLP* marked TCs in obesity (48). Gut-microbiota-derived succinate promotes TC increase and reduces intestinal inflammation (111). Both TCs and IL-25 decreased in obese patients and animal models (52). These decreases could be restored by berberine via the TAS2Rs Gα-gustducin/Gβ1γ13 signaling pathway (68). IL-25 was first identified as a cytokine that induced naive CD4^+^ T cells into CD4^+^ Th2 cells (116). It plays a crucial role in regulating Th2-type immunity by modulating actin related gene 1(*Act1*) (117) and also promoting the secretion of Th2 cytokines (74) (**Figure 2**). Studies on TCs, ILC2s and the circuit response to obesity are limited and more TCs models including organoids and knockout mice could be applied to investigate the mechanism of obesity barrier damage.

## 5 The TC-ILC2 circuit may be a potential intervention target in obesity

Obese mice induced by HFD were administered IL-25 for 21 days, their body weight, serum glucose and intraperitoneal glucose tolerance were remarkably reversed (118). Despite the circuit, Feng reported that IL-25 promotes M2 macrophage polarization and thereby stimulates lipolysis and mitochondrial activity against obesity (119). IPA supplementation prevents the development of HFD-induced obesity (52), and the dosage of IPA is negatively correlated with obesity-induced inflammation (120). IPA holds potential as a reagent for obesity treatment due to its ability to activate TCs and promote IL-25 production. Furthermore, it exhibits interaction with FFAR3, further highlighting its potential as a valuable tool in combating obesity (52). A recent study by Sun (68) discovered that berberine, when sensed by TAS2Rs in TCs, stimulates the synthesis of IL-25. This feedback in TCs may turn out to be a novel mechanism by which berberine ameliorates obesity. Supplementation of drinking water with succinate robustly suppressed weight gain and improved glucose tolerance in HFD mice, resulting in an elevation in global energy expenditure (121). Thus, intervention targets on TCs, ILC2s or the circuit including IL-25, IPA, berberine and succinate, dramatically reduced the obesity status.

## 6 Conclusion and prospects

TCs use apical microvilli to taste danger signals, particularly metabolites derived from worms, viruses and bacteria. In response, TCs trigger downstream pathways to produce cytokines such as IL-25 to promote ILC2 activity, leading to Lgr5^+^ stem cell differentiation into TCs in a positive feedforward loop. In obese subjects, the TCs, ILC2s and the circuit are disrupted. IL-25, IPA and berberine have been reported to rescue the altered TC-ILC2 circuit in obese subjects, yet the underlying mechanisms remain to be fully elucidated. TC knockout models (27) and organoids (68, 122) could be better tools for further revealing the mechanism of TCs and ILC2s in obesity.

## Author contributions

HY headed the team writing and editing. Y-XH, P-YX, J-QL, J-LC and XL, Y-JG contributed to writing and editing. W-JD reviewed and edited the draft. All authors contributed to the article and approved the submitted version.
